# 

**Published:** 1873-09

**Authors:** 


### *Books and Pamphlets.*

The absence from the city of the head of our review department must be our apology for omitting to notice a large number of books and pamphlets received during several weeks past. We shall endeavor in our next to make good all deficiencies in this regard, and notice all such seriatim and in detail. H.

